# Integrative medicine primary care: assessing the practice model through patients’ experiences

**DOI:** 10.1186/s12906-017-1996-5

**Published:** 2017-11-15

**Authors:** Robert L. Crocker, Amy J. Grizzle, Jason T. Hurwitz, Rick A. Rehfeld, Ivo Abraham, Randy Horwitz, Andrew Weil, Victoria Maizes

**Affiliations:** 1 University of Arizona Center for Integrative Medicine, College of Medicine, University of Arizona, Tucson, AZ USA; 2 Center for Health Outcomes & PharmacoEconomic Research (HOPE), College of Pharmacy, University of Arizona, Tucson, AZ USA

## Abstract

**Background:**

The University of Arizona Integrative Health Center (UAIHC) was an innovative integrative medicine (IM) adult primary care clinic in Phoenix, Arizona. UAIHC used a hybrid payment model to deliver comprehensive healthcare that includes conventional and complementary medical treatments.

**Methods:**

Fidelity measures were collected to evaluate how well the IM care delivery process matched ideals for IM. Patient experiences are presented here. Patients visiting UAIHC on 1 of 10 randomly selected days between September 2013 and February 2015 were surveyed. Patients were asked about their experience with: holistic care; promotion of health, self-care, and well-being; relationship and communication with practitioners; and overall satisfaction.

**Results:**

Eighty-three patients completed surveys. Based on patient-reported experiences, UAIHC delivered IM care as defined by the practice model.

**Conclusions:**

Patients received holistic care, established positive caring relationships with providers who promoted their self-care and well-being, and reported high overall satisfaction with UAIHC.

## Background

Integrative medicine (IM) is patient-centered, whole person healthcare embracing the body’s self-healing capacity and emphasizing the importance of lifestyle to enhance health (Maizes 2009; Rees 2001) [1, 2]. IM is an evidence-based, prevention-oriented, clinical approach that incorporates conventional medical treatment together with complementary medicine (CM) modalities (Boon 2004, Maizes 2002) [3, 4]. Components such as ready access to care, promotion of self-care, and good patient-practitioner relationship are central to achieving successful patient outcomes.

The University of Arizona Integrative Health Center (UAIHC), in Phoenix, Arizona, was a novel clinic offering integrative primary care. UAIHC was designed to embody integrative philosophies and an integrative care delivery model. In addition to the two fulltime University of Arizona Center for Integrative Medicine (AzCIM) IM fellowship-trained primary care physicians, practitioners included a chiropractor, 1–2 acupuncturists, a behavioral health clinician, a dietitian, a health coach and a nurse. Prior to opening the UAIHC, all staff members completed an online *Introduction to Integrative Medicine* course and participated in a 2-week training period. This training included introductory sessions in Motivational Interviewing and Mindfulness Based Stress Reduction, along with an in-depth overview of current literature on various integrative and complementary approaches to the treatment of several major common conditions, including diabetes mellitus, metabolic syndrome, cardiovascular disease and chronic pain. Although no specific guidelines were employed in care delivery, the staff were well trained in their perspective disciplines. UAIHC was supported by a hybrid financing structure that combines health insurance reimbursement with membership fees paid by patients and/or employers.

Key features of the model included completion of a detailed health intake by the IM physician in which all aspects of health and lifestyle were evaluated including but not limited to diet, sleep, activity, stress, relationships and spirituality. Each patient entered into a Health Partnership Agreement with their practitioner in which each individual and their physician committed to lifestyle change and personal responsibility. Care was delivered using a team care approached in which the health partnership philosophy was enacted in support of patients’ needs, and in a manner consistent with each patient’s goals, beliefs and values. Evidence on the potential impact of various treatment options and modalities offered in the health center were presented to patients, and individuals were supported in making informed decisions regarding which types of practitioners would be participating in their care team. A health coach was also available to aid individuals in successfully making and sustaining lifestyle change. There were also a number of groups and classes offered to patients at the health center including courses on nutrition, stress reduction, optimal weight and lifestyle, along with the opportunity to participate in yoga and Tai Chi groups offered on site.

During operation of the clinic, there were 1700 individuals who purchased memberships. The demographics of the clinic population closely matched those of the group that participated in this portion of the study and is described in the results section below.

While growing numbers of integrative clinics are being developed nationwide, few have tested whether an integrative model of care is actually being practiced. Therefore, it is challenging to assess which of the components of integrative medicine are being delivered, how well, and whether they contribute to health or cost outcomes (Dodds 2013) [5]. Fidelity refers to delivering an intervention as designed, here describing implementation integrity (Carol 2007) [6].

Research on UAIHC outcomes includes an assessment of fidelity to the principles and practices of IM using validated measures whenever possible; i.e., is the UAIHC practice model being delivered as intended? Is the care patient-centered? Do patients receive whole-person care? Do patients have access to care? The specific components included in the fidelity assessment are: patient-centeredness, whole person care, enhanced access to care, and patient satisfaction. Additional outcomes to be reported in other papers include patient reported outcomes of mental, physical, and overall health; work productivity and activity; and overall well-being.

### Patient-centeredness

Patient-centeredness encompasses practitioner communication style (listening, understanding, explaining, validating, empathy), patient-practitioner partnership (shared decision-making and treatment planning), adequate visit time, and patient trust (Stange 2010) [7]. Patient-centered care is generally assessed by the following areas of practitioner communication: 1) understanding the patient’s condition; 2) understanding the reasons for the visit and the patient’s information needs; 3) reaching a shared understanding of the treatment goals; and, 4) creating a continuing partnership (alliance) in which patients actively share in decision making and responsibility for their health. When these relational qualities are achieved, greater trust is instilled between patients and providers (Epstein 2005; Bertakis 2011) [8, 9]. Patients also may experience greater health self-efficacy (i.e., the extent to which patients feel capable of reaching their health goals) (Epstein 2005; Bertakis 2011) [8, 9]. Extended time with providers (60–75 min initial visits with 30-min follow-ups) allows sufficient time to discuss options and decisions, and allows the patient-practitioner relationship to grow (Maizes 2009) [1]. Questions from the Consumer Assessment of Healthcare Providers and Systems (CAHPS) (Hargraves 2003) [10] and the Consultation and Relational Empathy (CARE) measure (Mercer 2004) [11] were used to assess patient-centeredness.

### Whole person care

Whole person care requires attention to all patient factors influencing health, wellness, and disease, including body, mind, spirit, and community (Long 2002) [12]. These may encompass lifestyle choices, work and home environments, nutrition, interpersonal relationships, exercise and activities, and outlook on life. Items from the Ambulatory Care Experiences Survey (ACES) (Safran 2006) [13] and the CAHPS (Hargraves 2003) [10] evaluated whole person care.

### Enhanced access to healthcare

Access in primary care is the ease with which a patient can secure an appointment with a clinician without experiencing administrative and financial barriers (Bell 2002) [14]. The UAIHC hybrid financing approach was established as a means to increase patient access to healthcare through shorter wait times for appointments, same day appointments when clinically warranted, a broader primary care team, and longer appointments. For fidelity evaluation, access to care was assessed using a scale developed for the study that recorded the time between the patient’s initiation of service and receipt of an appointment. Items from the ACES (Safran 2006) [13] about courtesy and helpfulness of clinic front desk staff, and one item from the CAHPS (Hargraves 2003) [10] evaluated adequacy of the visit length and access.

### Patient satisfaction

Patient satisfaction ratings have long been used as dependent variables to evaluate health services and facilities on the assumption that satisfaction is an indicator of the structure, process, and outcomes of care. Satisfaction has also been used as an independent variable to predict patient behavior (e.g., service utilization, treatment adherence). Patient satisfaction was assessed with a 2-item measure from the CAHPS (Hargraves 2003) [10]. An additional measure of satisfaction was assessed by asking the likelihood of recommending UAIHC to family or friends.

## Methods

Following approval from the University of Arizona Institutional Review Board, questionnaires were developed to evaluate fidelity from both the patient, as well as the practitioner/staff perspective. Prior to initiating the study, a comprehensive literature review was conducted on integrative healthcare practice models, primary care clinic models, models of patient centered medical homes, and clinical and cost outcomes of these models. The literature was also reviewed on all *outcomes measures* to determine brevity, scoring algorithms, test-retest reliabilities and other psychometric properties, and use in the targeted population. Additionally, *fidelity measures* used by AHRQ (primary care) and the patient-centered medical home demonstration project were evaluated. Three thousand articles were identified matching some of the search criteria, and 850 were reviewed in detail. Although some of the articles looked at various aspects of integrative practice or the testing of tools within an integrative setting, no prior studies were found in which an integrative medicine model was the actual intervention. Hence, a fidelity study to measure intervention integrity was deemed a valuable contribution to the field of integrative medicine. The process used to identify the concepts for fidelity evaluation as well as a detailed description of the questionnaire development is reported elsewhere (Dodds 2013, Herman 2014) [5, 15].

### Patient assessment

Patient experience questionnaires contained 45 questions (Table 1) as well as demographic information. Questions employed dichotomous and Likert-response scales.

**Table 1:** Patient experience questionnaire

Topic area	# of Items	Description
Most recent visit	7	Assesses professionalism of front office personnel, wait times for scheduling appointments, coordination of care. Items rated on a 3-point scale from no; yes, somewhat; and yes, definitely.
Whole person care	4	Assesses being cared for as a whole person. Items rated on a 6-point scale from very poor, poor, fair, good, very good, excellent.
Health promotion	5	Assesses extent of promoting health, self-care, and well-being. Items rated as yes or no.
Relationship & communication	17	Assesses extent of feeling respected, trusting providers, spending sufficient time to discuss problems and action plans, and being involved in decision making. Items rated on a 3-point scale from no, yes, somewhat, and yes, definitely.
Practitioner empathy	10	From the *Consultation and Relational Empathy (CARE)* measure. Assesses extent of feeling that the practitioner really listened, understood, and cared; showed compassion and respect; and explained things clearly and honestly. Items rated on a 5-point scale from poor, fair, good, very good, excellent.
Overall satisfaction	2	Assesses satisfaction with UAIHC and willingness to recommend. Items rated on a scale from 1 (worst) to 10 (best).

To assess patient experience, self-administered questionnaires were completed by all patients consenting for the Fidelity Study who were seen on a randomly selected day of the week, ten separate times during the first two years of the study. Patients on these randomly selected days were approached in the waiting room and asked if they would be willing to complete the Fidelity questionnaire regarding their experience. These evaluations occurred monthly (beginning September 2013) for the first six months, quarterly for six months, then semiannually (ending February, 2015). Following their visit, patients were given the option to complete the questionnaire online or in a paper format. Those using the paper version could complete the survey at the clinic, return via postage-paid envelope, or return to a locked box at UAIHC. Patients opting for computer entry were sent a survey link with automated 3- and 6-day reminder emails if not completed. The study coordinator followed-up by phone with patients who did not submit a questionnaire within eight days. Similarly, the study coordinator was notified if mailed surveys had not been received. Patients completing the questionnaire within ten days received a $10 Amazon gift card.

### Analysis

Data from patient questionnaires were aggregated for descriptive analysis using Excel. Frequency of responses were reported by month of collection as well as an overall percentage for the entire study period. Means and standard deviations were calculated for the CARE measure: scoring ranges from 10 to 50, with higher scores being more favorable.

## Results

Eighty-three patients were approached in the waiting room on the randomly selected days and all completed surveys. Most were female (84%), white (82%), with over half (55%) between the ages 40–59 and (55%) married. Most were college educated (82%) and employed (74%). Complete respondent characteristics are included in Table 2.

**Table 2:** Demographic information

	Number	Percent
Gender		
Female	70	84.3%
Male	13	15.7%
Age		
18–39	12	14.6%
40–59	45	54.9%
60+	25	30.5%
Education		
High school	15	18.1%
College degree	33	39.8%
Graduate degree	35	42.2%
Employment status		
Employed	61	74.4%
Retired	15	18.3%
Other	6	7.3%
Race		
White	66	81.5%
Hispanic/latino	4	4.9%
Black	4	4.9%
Other	7	8.6%
Relationship		
Married/living together	56	67.5%
Single/divorced	19	22.9%
Divorced	8	9.6%

### Whole person care

Most patients felt practitioners had very good or excellent knowledge of their medical history (88%), values and beliefs about health (79%), worries and stress (69%), and their responsibilities at home or work (71%).

### Promotion of health

Most patients agreed that practitioners were promoting their health, self-care, and well-being by: talking with them about specific interventions to improve health (96%); giving help needed to change habits (95%) and maintain healthy body weight (76%); providing attention needed for emotional well-being (94%); and asking if their health status impacted activities of daily living (81%).

### Relationship and communication

All patients (100%) reported that practitioners treated them with respect. If discussing a health behavior change (*n* = 45), about 96% of patients were asked what they thought was best for themselves. Nearly all patients felt practitioners definitely spent enough time with them (98%). Patients felt practitioners really cared about them as a person (100% - 82% responded yes, definitely and 18% answered yes, somewhat), and cared about their health as much as they do (93% - 74% yes, definitely; 19% yes, somewhat). All patients trusted the practitioner with their care (84% definitely and 16% somewhat) and felt they would always tell them the truth (93% definitely and 7% somewhat). On a 10-point scale, about 93% of patients rated trust of their practitioner highly between 7 and 10. Table 3 summarizes the responses related to communication and relationship with the practitioner.

**Table 3:** Relationship and Communication with Practitioner (*n*, %)

	Yes, definitely		Yes, somewhat		No		Total
Practitioner showed respect for what patient had to say	79	97.5%	2	2.5%	0	0.0%	81
Practitioner spend enough time with patient	75	91.5%	5	6.1%	2	2.4%	82
Patient felt practitioner cares as much as they do about health	60	74.1%	15	18.5%	6	7.4%	81
Patient felt practitioner really cares about them as a person	66	81.5%	15	18.5%	0	0.0%	81
Patient trusted practitioner with their health care	69	84.1%	13	15.9%	0	0.0%	82
Patient felt practitioner would always tell truth about health	75	92.6%	6	7.4%	0	0.0%	81

### Empathy of practitioner

A summary of the CARE questions is included in Table 4. Total CARE scores range from 10 to 50, with higher scores reflecting more empathetic practitioners. Patients reported an overall mean (sd) score of 46.8 (5.3).

**Table 4:** Consultation and Relational Empathy (CARE) measure

Practitioner empathy questions	Mean (SD)
How was the practitioner at…	
1. Making you feel at ease	4.81 (.40)
2. Letting you tell your story	4.78 (.46)
3. Really listening	4.71 (.70)
4. Being interested in you as a whole person	4.59 (.75)
5. Fully understanding your concerns	4.61 (.80)
6. Showing care and compassion	4.73 (.53)
7. Being positive	4.85 (.46)
8. Explaining things clearly	4.69 (.70)
9. Helping you to take control	4.60 (.70)
10. Making a plan of action with you	4.51 (.66)
Total CARE score (range 10–50)	46.8 (5.3)

Responses based on a 5 point Likert scale from poor (1), fair (2), good (3), very good (4), excellent (5)

### Access to care - most recent visit

All patients (100%) felt the receptionist was respectful and courteous. About 62% of patients were able to get appointments in fewer than 5 days from when they wanted to be seen (with 42% getting appointments the same day or day following their request).

### Overall satisfaction

**Fig. 1:**
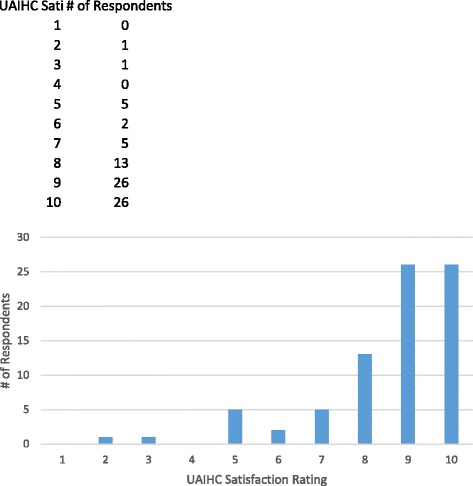
UAIHC satisfaction ratings, where 1 is the worst and 10 the best (*n* = 82)

On a scale of 1 (worst) to 10 (best), about 89% of patients rated overall satisfaction between 7 and 10 (see Fig. 1). Almost all patients (97%) would recommend the UAIHC to others.

### Limitations

The UAIHC fidelity assessment coincided with the opening of the clinic. As with the initiation of any new medical practice, the UAIHC clinic had challenges to overcome, which included implementing new electronic medical record and phone systems, dealing with new insurance procedures as part of the financial model, and establishing working relationships with all new clinic staff. This may have contributed to only 62% of patients getting care within five days. With this exception, and despite the challenges of start-up, overall satisfaction is extremely high and patient care did not appear to suffer.

While the demographics reported in this study may not reflect the average primary care practice in the United States, it is representative of the population seeking care at the health center at which this study was conducted.

Some UAIHC practitioners saw patients at the clinic every day of the week, while others practiced fewer days, so it is possible that interactions with some clinicians were underrepresented in the sampling. To account for this practice variation, patient experiences were solicited on a randomly selected day of the work week. However, the patients visiting the clinic on those days may not be reflective of all patients seen during the study period.

Although patients were assured that their responses were anonymous, they may have felt their identity could be traced through their email address. This could have led to social desirability bias, where the respondent answers what they believe the investigator wants to hear.

The model emphasizes team-based care and patients were queried after seeing any clinician or after an educational class or group visit. This makes it hard to assess which of the providers may have had the most influence.

Another limitation is the small sample size.

## Discussion

The specific components assessed for this fidelity evaluation included: patient-centeredness, whole person care, enhanced access to care, and patient satisfaction.

Research has demonstrated the importance of patient-provider relationships and the impact it can have on patient outcomes (Fenton 2012; Boulding 2011; Glickman 2010) [16–18]. Typically, primary care visits are scheduled for fifteen minutes, despite the variation in complexity a patient may present (Margolius 2010) [19]. It is nearly impossible for a practitioner to develop a meaningful relationship with a patient, listen attentively to the patient’s concerns, and engage them in developing an effective treatment plan within the confines of a fifteen-minute appointment. The additional time carved out for patient visits is a crucial element of the IM primary care model. The positive responses related to patient-centeredness and whole-patient care may be significantly attributed to the extended visit length and what is achieved during that time. These results are corroborated by findings from Adoph et al. showing patient satisfaction was highly correlated with the length of primary care visit (Adoph 2011) [20]. For patients spending more than 10 min with their primary care provider, the likelihood of a satisfactory rating increased dramatically (OR, 82.0, 95% CI, 64.8–103.8; *P* < .001).

However, length of visit is not the only important factor; the fidelity results also point to the quality of the interaction with the provider. In this study, patients reported extremely positive scores for provider empathy, listening, caring and trust. These are central values to integrative medical care. Several studies connect physician empathy with enhanced health outcomes. A study of exemplary family physicians (who had additional training in counseling) revealed enhanced emotional support and more patient involvement in decision making with no additional time spent during appointments (Marvel 1998) [21]. A study published in *Family Medicine* (Rakel 2009) [22] correlated improved outcomes for patients with a common cold (decreased cold severity and shortening of cold duration) who perceived that their physician exhibited high levels of empathy as measured by CARE scores. A study of German cancer patients (Neumann 2007) [23] demonstrated improved quality of life and a preventive effect on the development of depression among patients who perceived greater levels of empathy in their physicians. Although more research is needed on the relationship between empathy and patient outcomes, these studies demonstrate that the quality of physicians’ interactions with their patients may be of clinical importance.

We evaluated enhanced access to care via wait times for obtaining appointments. About 62% of patients were able to get appointments fewer than 5 days from their request, with 42% getting appointments the same day or day following their request. These relatively short wait times likely contributed to the high patient satisfaction ratings. These results are consistent with those found in a primary care study surveying over 11,000 patients, which showed satisfaction was significantly higher when appointments were scheduled within 2 days of calling compared to waiting 3 to 5 days (odds ratio [OR], 0.46; 95% confidence interval [CI], 0.39–0.53; *P* < .001) (Adoph 2010) [20].

In addition, this practice placed significant emphasis on the promotion of health. Patients reported that practitioners discussed specific interventions to improve health and helped them to change habits. Given the high burden of chronic illness in the US, the high rate of attention to lifestyle change and shared decision making likely contributed to the high satisfaction of the patients.

These findings are similar to those reported by the University of Michigan Integrative Medicine Clinic (Myklebust 2008) [24]. Over 60% of the 274 patients surveyed rated their care as “excellent” or “best care ever” (37.6% and 24.7%, respectively). Others rated their experiences as “above average” (16.5%), “good” (11.8%), or “poor” (7.1%). A study by Holland et al. (Holland 2016) [25] found that shared decision making, a principal component of IM, led to higher patient satisfaction in 94 older adults seen in the emergency department for musculoskeletal pain.

Not evaluated in this study, but nonetheless an important question, is what role practitioner and staff training may play in improving patient outcomes. For many years, healthcare educational institutions and regulatory bodies have set minimum standards for practitioner education, in part to promote safer and higher quality care. At the UAIHC, additional educational training was required (or provided), including IM fellowship training for primary care practitioners, and the described 2-week training provided for all staff members prior to opening. Although not measured in this study, whether or not there is a link between staff training and patient outcomes in an integrative primary care setting may well be worthy of study.

## Conclusion

US public satisfaction with the health care system has been lower than in other high-income countries for several decades (Hero 2016) [26]. Many believe the system is broken, and there have been myriad suggestions to remedy the problem. Based on our study, an IM primary care approach may contribute to the solution. Not only is patient satisfaction high, health promotion and emotional well-being are also addressed. UAIHC patients were pleased to recommend the UAIHC to others.

Based on patient-reported experiences, UAIHC delivered IM care as defined by the practice model. Patients received whole person care, established positive caring relationships with providers who promoted their self-care and well-being, and reported high overall satisfaction with UAIHC. These findings speak strongly in support of models that are integrative, team-based and truly patient centered. Additionally, these results have implications relative to improving patient satisfaction and potentially outcomes not just for the field of integrative medicine but for the health care system in general.

## Abbreviations

ACES: Ambulatory Care Experiences Survey
AzCIM: Arizona Center for Integrative Medicine
CAHPS: Consumer Assessment of Healthcare Providers and Systems
CARE: Consultation and Relational Empathy
CM: Complementary Medicine
IM: Integrative Medicine
UAIHC: University of Arizona Integrative Health Center
